# Research on SAR Image Target Recognition Method Based on Multi-Dimensional Feature Fusion

**DOI:** 10.3390/s26123677

**Published:** 2026-06-09

**Authors:** Jiaqi Fang, Hemin Sun, Hongquan Li

**Affiliations:** Air Force Early Warning Academy, Wuhan 430019, China

**Keywords:** SAR images, target recognition, feature extraction, hierarchical extraction, adaptive weighting, multi-dimensional fusion

## Abstract

Synthetic Aperture Radar (SAR) has broad application prospects in target recognition; however, intrinsic multiplicative speckle noise, geometric distortions, and the complex coupling of multimodal features often limit the comprehensiveness of representations and the efficiency of fusion, thereby restricting recognition accuracy. To address these limitations, this paper proposes a SAR image target recognition method based on multidimensional feature fusion. The proposed method first achieves noise suppression and contrast enhancement through an optimized preprocessing layer. Subsequently, a dual-branch hierarchical feature extraction network synchronously captures low-dimensional physical prior features driven by domain knowledge and highly discriminative deep convolutional features, ensuring a balance between physical interpretability and high-capacity representation. Finally, a variance-adaptive weighted fusion layer dynamically balances the contribution of different feature streams, mitigating information redundancy and feature conflict. Quantitative experiments on the MSTAR and public CETC38-SAR datasets demonstrate that under various pre-trained backbones, the proposed framework improves precision, recall, and F1-score by 5%–15% compared with baseline methods. Ablation studies and evaluations under extended operating conditions further validate the robustness, computational efficiency, and structural validity of the decoupled architecture.

## 1. Introduction

Synthetic Aperture Radar actively emits electromagnetic waves and receives scattered echoes from targets, enabling it to penetrate complex environments such as clouds, fog, and vegetation to obtain high-resolution images. It plays an irreplaceable role in geological survey, climate monitoring and military reconnaissance applications [1,2,3,4]. However, affected by speckle noise, multiple scattering, and geometric distortions, target features in SAR images exhibit nonlinear and multimodal coupling characteristics, rendering conventional feature extraction approaches tailored for optical imaging invalid for SAR interpretation [5]. Therefore, effective analysis and interpretation of target features in SAR images are the core of achieving battlefield target detection and identification.

To fully exploit the potential of intelligent interpretation, deep learning-based convolutional neural networks (CNNs) and Vision Transformers have gradually become the mainstream paradigm for SAR object detection. Early studies directly transplanted mainstream optical detection architectures including Faster R-CNN and YOLO series into SAR interpretation tasks, but these unconstrained models often suffer from high false-alarm rates due to strong land clutter and speckle noise. Consequently, recent advanced algorithms have begun incorporating SAR-specific physical or environmental priors. For instance, Ke et al. proposed the Sea–Land Aware Network (SLA-Net), which introduces a sea-land prior-guided hierarchical attention mechanism [6]. By coupling a land–sea segmentation branch with an attention-driven Feature Pyramid Network (FPN), SLA-Net dynamically suppresses onshore vehicle/building interference and concentrates the representation capacity purely on maritime regions, significantly improving small ship detection performance in complex coastal areas.

In-depth mining and analysis of target features are prerequisites for rapid detection, accurate recognition, and precise classification, which are crucial for real-time battlefield situation awareness, rational allocation of combat resources, and scientific strategy formulation. In recent years, numerous researchers have conducted extensive research on target feature extraction for SAR images. Ai et al. designed a Multi-scale Rotation-invariant Haar-like Feature Integrated CNN (MSRIHL-CNN). This method combines multi-scale rotation-invariant Haar-like low-level features with high-level deep features, effectively capturing local texture and edge information that is easily lost in conventional CNNs, and achieving excellent detection performance in multi-target SAR scenes [7]. On this basis, Ai et al. further proposed a Multi-kernel-size Feature Fusion-based CNN (MKSFF-CNN). By constructing a multi-channel parallel topology with convolutional kernels of different sizes, the model extracts multi-scale deep features and performs optimal fusion, significantly improving the completeness of SAR target feature representation and obtaining superior classification results on the MSTAR dataset [8]. Xue et al. proposed a Lightweight Modality Compensation Network (LMCNet) via knowledge distillation. Aiming at the problem of missing modalities in practical applications, this method realizes effective compensation of missing modal information through lightweight knowledge distillation, and achieves a good balance between detection accuracy and computational efficiency, which is suitable for real-time interpretation tasks on resource-constrained platforms [9]. Long et al. proposed a sparse representation parameter estimation method fusing image domain information to reduce the temporal and spatial complexity of the algorithm, achieving accurate extraction of SAR attributed scattering centers [10]. Zhang et al. proposed a SAR image target recognition method based on deformable convolutional neural networks, enhancing the network’s ability to extract salient features in target regions and effectively adapting to geometric distortion in images [11]. Jiang et al. proposed the SARCLIP multimodal framework to meet the demand for multimodal feature representation of SAR images, realizing cross-modal feature fusion of SAR images through contrastive learning and expanding the dimension and generalization ability of feature representation [12]. Xue et al. proposed a rotated target detection method based on global feature fusion, improving detection accuracy by integrating multi-feature fusion and rotated feature alignment [13]. Zhao et al. significantly improved small-target detection accuracy by designing the lightweight XMNet model that balances feature representation capability and real-time performance [14,15].

Advancements in the above deep-learning-driven SAR detection algorithms heavily rely on the construction of standardized benchmark datasets. Among them, the SAR Ship Detection Dataset (SSDD) released by Li et al. and formally institutionalized by Zhang et al. stands as the pioneering open-source dataset in this community, providing multi-sensor, multi-polarization (HH, VV, VH, HV), and multi-resolution (1 m to 15 m) SAR imagery for bounding-box and rotated-box regression [16]. To bridge the gap between small cropped slices and practical large-scene space-borne applications, Zhang et al. further introduced the Large-Scale SAR Ship Detection Dataset (LS-SSDD-v1.0) [17]. Sourced from Sentinel-1, LS-SSDD-v1.0 preserves 15 massive original SAR swaths cut into 9000 sub-images, encompassing extensive pure land backgrounds and complex inshore environments, which poses a severe challenge for balancing detection speed and false alarm suppression.

At the same time, with the introduction of attention mechanisms, capsule networks, and Transformer architectures into SAR image target detection and recognition (SAR ATR), the model’s adaptability to SAR noise, geometric distortion, and small-sample scenarios has been significantly improved. Huo et al. integrated convolutional attention with capsule networks to effectively capture hierarchical spatial features under few-shot conditions, improving recognition robustness [18]. Zhao et al. proposed a channel attention-based few-shot SAR aircraft classification method, which mitigated feature redundancy and noise interference by enhancing weights of critical feature channels [19]. Yu et al. constructed an attention-driven lightweight SAR ship detector, which reduced model parameters while maintaining accuracy, making it suitable for real-time monitoring [20]. With the widespread adoption of Transformers in vision tasks, Beal et al. explored Transformer-based object detection frameworks, verifying their potential for global modeling without convolutions [21]. Hou et al. combined multi-scale capsule structures with Swin Transformer to model global–local feature interactions, enhancing long-range dependency modeling for SAR targets in complex scenes [22]. Wang et al. further proposed the Pyramid Vision Transformer (PVT), a multi-scale hierarchical architecture adapted for dense prediction tasks, offering new insights for global SAR feature extraction [23].

The above methods have advanced research on SAR target feature extraction from various fields and perspectives. However, existing attention mechanisms often focus on a single feature domain, making it difficult to simultaneously ensure the integrity and robustness of feature representation, with weak anti-interference capability and lacking physical constraints. Multimodal fusion methods rely too heavily on data scale, resulting in limited generalization. Transformer-based models, while strong in global modeling, have high computational costs and insufficient adaptability to small-sample SAR scenarios. Additionally, existing methods are inadequate in adapting to multi-scale and complex scenarios, and issues of information loss and redundancy in cross-level feature transfer have not been fully addressed. Furthermore, most methods perform poorly in balancing positioning accuracy and noise suppression, making it difficult to maintain accurate target positioning while suppressing coherent speckle noise and background clutter.

It is worth noting that while modern end-to-end target detection paradigms (such as YOLO series, Faster R-CNN, and DETR) have achieved great success in optical domains, their direct deployment in SAR applications is often hindered by intense multiplicative speckle noise, severe geometric distortions, and the scarcity of large-scale labeled datasets. End-to-end models attempting to simultaneously optimize localization and classification boundaries without explicit physical constraints are more susceptible to local overfitting and high false-alarm rates. Therefore, decoupling the task into a robust localized preprocessing stage followed by an advanced multi-dimensional feature-fused recognition network is a highly rational and strategic paradigm for complex SAR scenarios.

To address the problems of one-sided single-feature representation, inefficient multi-dimensional feature fusion, insufficient adaptability to complex scenarios, and difficulty in collaborative optimization of noise suppression and positioning accuracy in current SAR image target feature extraction, this paper proposes a SAR image target recognition method based on multidimensional feature fusion. By constructing a multi-dimensional feature collaborative representation mechanism, designing a dual-branch hierarchical feature extraction architecture, and building an adaptive weighted fusion layer driven by dual-channel attention, it achieves complementary representation and deep integration of multi-dimensional features, as well as the collaborative unification of physical feature interpretability and deep feature strong representation capability. Experimental results show that the proposed method effectively improves the comprehensiveness of SAR image target feature extraction and the accuracy of target recognition, providing methodological support for subsequent research on target recognition based on SAR images.

## 2. Analysis of Target Feature Extraction Methods

**Figure 1:**
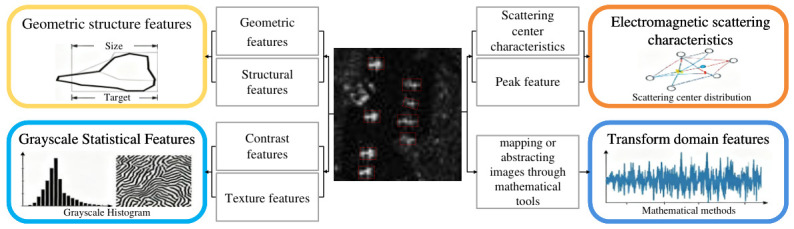
Typical features of SAR images.

SAR images differ significantly from optical images. Affected by the imaging mechanism, they cannot fully present target shapes, mainly showing sparse scattering center distributions. Moreover, target representations vary with azimuth angles under different imaging conditions. Typical features of SAR images, as shown in Figure 1, can be divided into geometric structure features, gray statistical features, electromagnetic scattering features, and transform-domain features according to different representation forms. These features describe the physical geometric structure, gray distribution characteristics, electromagnetic scattering parameters, and other information of targets, respectively [24]. Effectively analyzing SAR image features and extracting information that stably describes targets is the key to improving the efficiency of battlefield target detection and recognition.

The core of geometric structure feature extraction is to restore the real spatial features of targets from nonlinear distortions in SAR images, mainly using edge detection, contour fitting, morphological analysis, and other methods [25]. However, edge detection algorithms are vulnerable to speckle noise and thus susceptible to edge fracture and spurious edge generation; contour fitting has high computational complexity and is sensitive to initial contour positions, making it difficult to meet real-time battlefield requirements; morphological analysis relies excessively on preset structural elements and has insufficient ability to describe details of complex targets.

The key to gray statistical feature extraction is to mine pixel gray distribution rules and spatial correlations, commonly using histogram analysis, Gray-Level Co-occurrence Matrix (GLCM), and other methods [26]. Histograms can quickly achieve initial segmentation of targets and backgrounds but cannot reflect spatial correlations or texture details of pixels; GLCM-based extraction demands empirical parameter tuning, leading to drastically elevated computational overhead for high-resolution SAR images, making it difficult to adapt to complex combat scenarios.

Extracting electromagnetic scattering features requires analyzing target physical parameters from scattered echoes, mainly using scattering center modeling and peak detection [27]. Scattering center modeling can be associated with target physical structures and has strong interpretability but is significantly affected by azimuth angles, requiring re-modeling after target attitude adjustment; peak detection has high computational efficiency and is suitable for rapid screening of candidate target regions but easily produces false peaks in SAR images due to multiple scattering, leading to an increased false alarm rate.

Transform-domain feature extraction is mainly based on mathematical mapping to mine high-dimensional implicit features of images, commonly using Principal Component Analysis (PCA), Independent Component Analysis (ICA), Wavelet Transform (WT), and other methods [24]. PCA can reduce dimensions and remove redundancy but easily loses target feature information; ICA can separate independent signals from noise but has insufficient adaptability to complex scattering mechanisms; Wavelet transform achieves denoising yet depends on artificial basis function screening, which limits its generalization performance, and traditional methods lack translation invariance, easily introducing pseudo-Gibbs effects.

## 3. Design of Multi-Feature Extraction Method for Targets

Single-feature extraction methods can only describe targets from a specific perspective, and detection and recognition performance may be limited when facing complex and changeable environments and diverse targets. To address the fragmentation of feature dimensions in SAR images and the inability of single features to meet target detection and recognition requirements, multi-domain feature fusion of target geometric shape, material characteristics, electromagnetic scattering, and other aspects can be performed to solve the problem of feature dispersion in traditional feature extraction.

**Figure 2:**
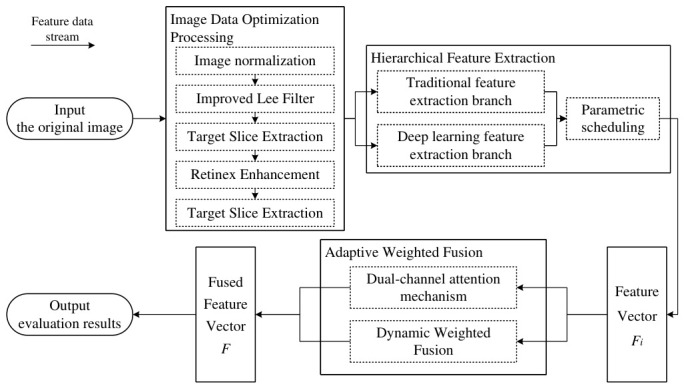
Multi-dimensional collaborative representation method for SAR image target feature fusion and extraction.

This paper proposes a multi-dimensional collaborative representation method for SAR image target feature fusion and extraction. It optimizes image data through image normalization, data augmentation, Retinex enhancement, and target slice extraction. A hierarchical architecture is designed to combine traditional feature extraction with deep learning-based feature extraction, leveraging the advantages of multi-dimensional target features. By utilizing channel/spatial dual-channel adaptive weighted fusion to compute feature vectors. The method achieves a more comprehensive and accurate description of target features, further enhancing the accuracy and generalization capability of target recognition. Unlike fully unconstrained end-to-end architectures, the proposed method intentionally adopts a “pre-processing/slicing + multi-dimensional feature representation + adaptive classification pattern”. This decoupled design allows the network to bypass the heavy background clutter and focus its representation capacity entirely on the structural scattering centers of the targets, striking a balance between statistical learning and physical domain knowledge. The system architecture and feature data stream of this method are shown in Figure 2.

### 3.1. Image Data Optimization Processing Layer

The image data optimization processing layer first calculates local statistics of the original SAR image. Following the fundamental principle of the classic Lee filtering algorithm, it optimizes filtering coefficients by introducing edge detection operators, preserving target edge information while suppressing speckle noise [28]. The improved Lee filter algorithm can be expressed as

(1) $$\hat{I}\left(x,y\right)=\mu +\frac{\sigma_{s}^{2}}{\sigma_{s}^{2}+\sigma_{n}^{2}}\left[I\left(x,y\right)-\mu \right]$$

where $\hat{I}\left(x,y\right)$ represents the denoised SAR image; μ and $\sigma_{s}^{2}$ represent the mean value of the noisy image and the variance of the real signal in the local window, generally estimated through window statistics; $\sigma_{n}^{2}$ denotes noise variance, which can be empirically predefined or statistically calculated from homogeneous image patches.

This layer enhances SAR image feature data using strategies such as rotation, scaling, translation, and flipping, and designs data enhancement configuration modes including standard enhancement, aggressive enhancement, geometric transformation, noise enhancement, and minimal enhancement to adapt to SAR image data enhancement requirements under different imaging conditions.

In addition, based on typical features of SAR targets, this layer combines Retinex theory on the basis of the improved Lee filter. It first estimates the illumination component using Gaussian blur, then separates the reflectance component through ratio calculation to enhance target detail contrast [29]. The Retinex enhancement method can be expressed as

(2) $$L\left(x,y\right)=G_{\sigma }\left(x,y\right)*I\left(x,y\right)$$

(3) $$R\left(x,y\right)=\frac{I(x,y)}{L(x,y)+\varepsilon }$$

where $G_{\sigma }\left(x,y\right)=\frac{1}{2\pi \sigma^{2}}e^{-\frac{x^{2}+y^{2}}{2\sigma^{2}}}$ is the Gaussian kernel; ∗ represents the convolution operation; I(x,y) is the SAR image after Lee filter denoising; R(x,y) is the reflectance component containing target details; $\varepsilon =10^{-8}$ is a small value to avoid division by zero. Meanwhile, this layer uses the Otsu algorithm to adaptively segment targets and backgrounds [30], removes noise points using morphological operations, extracts target slices combined with contour moment calculation, and finally realizes functions such as image normalization, data enhancement, and target slice extraction.

### 3.2. Hierarchical Feature Extraction Layer

**Figure 3:**
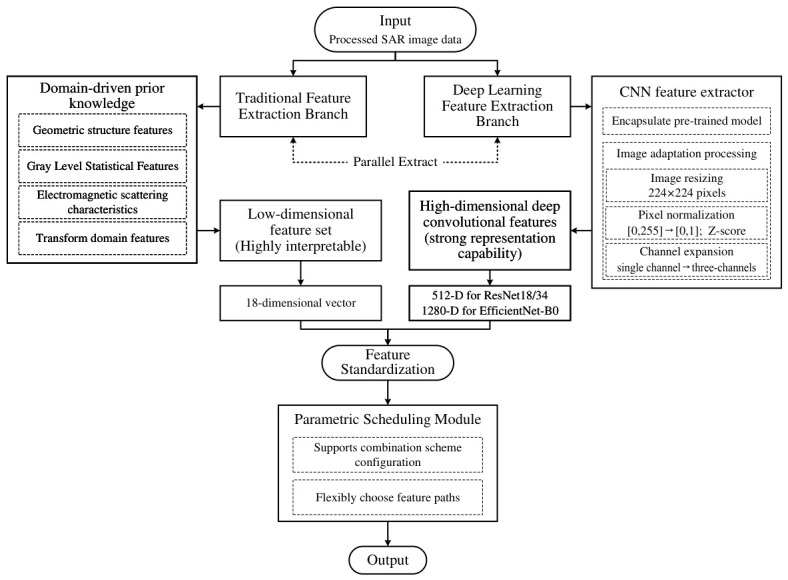
Hierarchical feature extraction layer.

The hierarchical feature extraction layer adopts a design concept of multi-dimensional representation, dual-path parallelism, and high-adaptability output, integrating the extraction capabilities of traditional methods and deep learning. As shown in Figure 3, the main body adopts a dual-branch hierarchical architecture. One branch is the traditional feature extraction branch, which conducts parallel multi-dimensional feature extraction on preprocessed SAR images through hierarchical design driven by domain prior knowledge, comprehensively mining different types of target feature information and generating corresponding feature vectors to form a low-dimensional feature set with strong interpretability. The second is the deep learning feature extraction branch. First, the input images are uniformly resized to 224×224 pixels through image scaling. The pixel range of the single-channel images is linearly mapped from [0,255] to [0,1], followed by Z-score normalization. To meet the input requirements of the pre-trained models, Single-channel normalized grayscale maps are duplicated to form three-channel input tensors to match pre-trained network requirements, ultimately completing the adaptive processing of the SAR images. Finally, a CNN feature extractor is constructed to encapsulate pre-trained models such as ResNet18/34 [31] and EfficientNet-B0 [32] to extract high-dimensional deep convolutional features with strong representation capabilities.

Among them, Z-score normalization can be defined as

(4) $$I_{norm}=\frac{I\left(x,y\right)-\mu_{SAR}}{\sigma_{SAR}}$$

where $I_{norm}$ is the normalized image pixel; $\mu_{SAR}$ is the global mean of SAR image pixels in the training set; $\sigma_{SAR}$ is the global standard deviation of SAR image pixels in the training set.

This layer flexibly selects feature paths or combination schemes through parametric scheduling of the system framework. The traditional feature branch provides basic features for the fusion logic of hierarchical extraction and dynamic weighting, while the deep learning feature branch forms a performance comparison with the traditional scheme. The extracted features, after standardization, can be directly connected to the dynamic weighted fusion layer, realizing end-to-end connection from feature extraction to optimized output. This not only ensures the comprehensiveness and pertinence of features but also provides comparative support for verifying the effectiveness of the method through dual-path design.

### 3.3. Adaptive Weighted Fusion Layer

**Figure 4:**
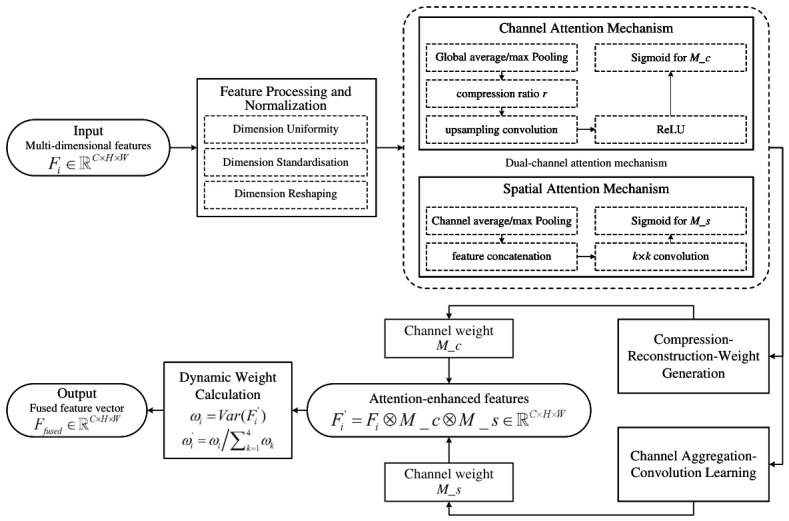
Adaptive weighted fusion layer.

The adaptive weighted fusion layer integrates channel attention, spatial attention, and a dynamic weight calculation mechanism to achieve adaptive enhancement and optimized integration of SAR image target features, with the design concept shown in Figure 4. This layer uniformly maps input multi-dimensional features to the target dimension, solves dimension mismatch through padding or truncation, and replaces null or abnormal features with zero vectors to ensure input consistency and effectiveness. Meanwhile, it uses a dual-channel attention mechanism to strengthen key feature information, outputs channel weights M_c and spatial weights M_s through convolutional layer compression, reconstruction, and spatial dimension learning, and enhances SAR target feature maps through dual-channel attention weights to obtain attention-enhanced features $F_{i}^{\prime }$ [33]. The attention-enhanced features $F_{i}^{\prime }$ can be summarized as

(5) $$F_{i}^{\prime }=F_{i}⊗M\_c⊗M\_s$$

where $F_{i}\in R^{C\times H\times W}$, *C* is the number of feature channels, *H* and *W* are the dimensions of the SAR target feature map, ⊗ represents broadcast multiplication for element-wise multiplication.

The mechanism for generating dynamic weights is based on information entropy and statistical variance principles. For a given attention enhancement feature tensor $F_{i}^{\prime }$ channel variance and spatial variance, the dispersion of active semantic responses can be quantified. Formally, a larger feature variance indicates that the corresponding feature branch carries richer discriminative information and distinct target boundaries, while smaller variance indicates uniform activation distribution, usually corresponding to background noise or redundant characterization.

In the dynamic weighted fusion stage, the dual-channel attention mechanism is used to adaptively calculate dynamic weights, and weighted summation is performed on the dynamic weights and four types of feature vectors to obtain fused feature $F_{fused}$. The calculation process of fused feature $F_{fused}$ can be summarized as

(6) $$\omega_{i}=Var\left(F_{i}^{\prime }\right)$$

(7) $$\omega_{i}^{\prime }=\omega_{i}/\sum_{k=1}^{4}\omega_{k}\;$$

(8) $$F_{fused}=\sum_{i=1}^{4}\omega_{i}\cdot F_{i}^{\prime }$$

where Var(⋅) is the variance of the feature vector, The dynamic weight $\omega_{i}^{\prime }$ is a normalized expression of $\omega_{i}$, $\sum \omega_{i}=1$, i=1,2,3,4.

The channel attention calculation mechanism uses global pooling for information statistics, then completes channel weight calculation through convolutional layer compression, dimensionality reduction, and weight activation. The weight value represents the richness of target information in the corresponding channel of the SAR image. The channel weight calculation process is specifically expressed as

(9) $$F_{avg}^{c}=\frac{1}{H\times W}\sum_{i,j}F_{c,i,j};F_{\max}^{c}=\max_{i,j}F_{c,i,j}$$

(10) $$F^{rec}=conv_{k\times k,C/r\;\to C}\left(\mathrm{ReLU}\left(conv_{k\times k,C\to C/r\;}\left(F^{c}\right)\right)\right)$$

(11) $$M\_c=\sigma (F_{avg}^{rec}+F_{\max}^{rec})$$

where $F_{avg}^{c}$ and $F_{\max}^{c}$ represent the global average pooling feature and maximum pooling feature, respectively; $F^{rec}$ represents channel compression and dimensionality reduction through convolutional layers, balancing computational cost and representation capability, and capturing nonlinear dependencies between channels; *r* is the channel reduction ratio; $conv_{k\times k,in\to out}$ represents a convolutional operation with kernel size is k×k, input channels is in, and output channels is out, followed by ReLU activation to enhance nonlinearity; $\sigma \left(\cdot \right)=\frac{1}{1+e^{-\cdot }}$ is the sigmoid function.

The spatial attention calculation mechanism compresses spatial position information through channel pooling, then completes spatial weight calculation through convolutional fusion and weight activation. The weight value represents the probability that the corresponding spatial position is the target region in the SAR image. The spatial weight calculation process is specifically expressed as

(12) $$F_{avg}^{s}=\frac{1}{C}\sum_{c=1}^{C}F_{c,i,j};F_{\max}^{s}=\max_{c=1\to C}F_{:,c,i,j}$$

(13) $$M\_s=\sigma (f^{k\times k}\left[F_{avg}^{S},F_{\max}^{S}\right])$$

where $F_{avg}^{s}$ and $F_{\max}^{s}$ represent compressing spatial position information using channel average pooling and maximum pooling, respectively; $f^{k\times k}$ represents a convolutional operation with kernel size.

The adaptive weighted fusion layer enhances feature discriminability through the attention mechanism, adapts to the contribution of different features through dynamic weights, effectively improves the fusion efficiency of multi-source features, provides more representative input features, and significantly enhances the adaptability and robustness of the method to complex scenarios.

Unlike traditional dual-branch attention fusion schemes, the proposed method emphasizes cross-modal collaborative guidance and dispenses with the conventional practice of utilizing fixed or learnable fusion weights directly after feature concatenation. In the adaptive weighted fusion layer, the spatial and channel weights of dual-channel attention are first leveraged to enhance feature discriminateness, followed by the calculation of dynamic weights based on the variance of the attention-enhanced features. Furthermore, the proposed dual-branch architecture is guided by SAR physical scattering characteristics rather than mere dataset statistics. The preprocessing module is co-optimized with the downstream feature extraction, thereby constructing a full-link fusion design that distinguishes itself from existing isolated dual-branch structures.

## 4. Experiments and Analysis

### 4.1. Experimental Data Selection and Indicator Quantification

**Figure 5:**
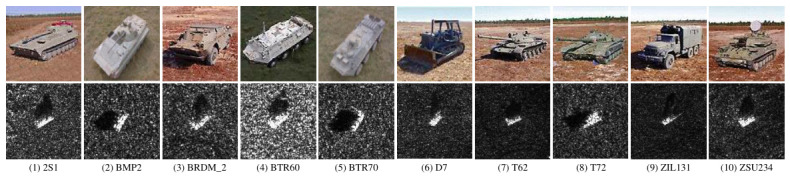
Optical and SAR images of 10 types of targets in the MSTAR dataset.

**Figure 6:**
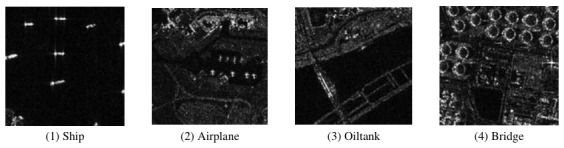
SAR images of 4 types of targets in the CETC38-SAR dataset.

This paper mainly uses two types of SAR image datasets. One is the MSTAR dataset released by DARPA/AFRL of the United States [34,35], which includes 10 categories of X-band target images (2S1, BMP2, BRDM_2, BTR60, BTR70, D7, T62, T72, ZIL131, ZSU234) with resolution and pitch angles of 15° and 17°. The other is the CETC38-SAR dataset released by the 38th Research Institute of China Electronics Technology Group Corporation, which includes 4 categories of target images (ships, aircraft, oil tanks, bridges), consisting of 13,038 ships, 5699 aircraft, 919 oiltanks, and 551 bridges. Figure 5 and Figure 6 show examples of target images in the two datasets.

In the field of SAR image target feature research, precision (Pre), recall (Rec), and F1-score are mainly used to test the availability of target features and the effectiveness of extraction methods. Precision quantifies the proportion of truly positive samples among all predicted positive candidates, which is widely adopted as detection accuracy in target recognition tasks. Recall refers to the ratio of correctly detected and recognized targets to the actual number of targets, i.e., “check completeness”. F1-score is a comprehensive indicator evaluating classification performance by considering both precision and recall [36,37].

The specific definitions are as follows:

(14) $$Pre=\frac{TP_{m}}{TP_{m}+FP_{m}}$$

(15) $$Rec=\frac{TP_{m}}{TP_{m}+FN_{m}}$$

(16) $$F1-score=2\times \frac{Pre\times Rec}{Pre+Rec}$$

where $TP_{m}$ represents the number of targets correctly predicted as the *m*-th category; $FP_{m}$ represents the number of targets incorrectly predicted as the *m*-th category; $FN_{m}$ represents the number of targets actually belonging to the *m*-th category but incorrectly predicted.

In terms of efficiency performance evaluation, the parameter size, floating-point operations, and inference time are adopted as verification indicators. Among them, Parameter Size (Params) refers to the total number of parameters of the trained model, which reflects the spatial complexity of model computation, with the unit of millions (M). Floating Point Operations (FLOPs) serve as a metric to measure the complexity of an algorithm or model, representing the total number of floating-point operations required during the execution process of the algorithm or model. Inference Time denotes the average time taken for a single SAR image from the initial input to the final output of detection and recognition results, with the unit of seconds per image (s/img).

### 4.2. Experimental Setup

Based on the characteristics of the MSTAR and CETC38-SAR datasets, as well as the requirements for target feature extraction, detection, and recognition, the datasets are split into training and test sets at an 8:2 ratio, with a fixed random seed of 42 to ensure experimental reproducibility. In the training process, a pre-trained mode was used to determine the weights, and no parameter adjustments were made in subsequent stages. In the feature extraction stage, on one hand, features including geometric structure, gray-level statistics, electromagnetic scattering, and transform-domain features were extracted and concatenated into an 18-dimensional feature vector; on the other hand, three convolutional neural networks—ResNet18/34, and EfficientNet-B0—were used to extract deep features, producing 512-dimensional or 1280-dimensional deep features, with a feature extraction batch size set to 6. Meanwhile, for the Gaussian noise gradient in the experiment’s noise robustness test, the range was set to 0.01–0.10.

In the pre-training mode, ResNet18/34 both used the SGD (Stochastic Gradient Descent) optimizer, with momentum set to 0.9, weight decay set to 0.0001, and learning rate using a Step decay strategy. The initial learning rate was set to 0.1, batch size to 256, and pre-training lasted for 90 epochs. EfficientNet-B0 used MBConv as the backbone, with composite scaling factors for depth, width, and resolution set to 1.0, 1.0, and 224 respectively, and used the RMSProp (Root Mean Square Propagation) optimizer, with momentum set to 0.9, weight decay 0.00001, and learning rate following a Cosine Annealing strategy. Its initial learning rate was set to 0.256, batch size to 256, and pre-training lasted for 350 epochs.

In the deep learning feature extraction branch, ResNet18 and ResNet34 are intentionally selected as representative backbones rather than deeper architectures like ResNet50 or ResNet101. This selection is guided by three primary considerations:

1. Avoiding overfitting on small-sample SAR imagery. Unlike large-scale optical classification datasets (e.g., ImageNet), public SAR target datasets such as MSTAR contain relatively sparse sample sizes with limited aspect angles. Deploying an overly complex backbone like ResNet50 with vast parameters significantly increases the risk of semantic overfitting and memory saturation.
2. Alignment with the lightweight task requirements. Real-time battlefield situation awareness requires a strict balance between feature representation capability and computational efficiency.
3. Coordination with our multi-dimensional collaborative architecture. Since our framework explicitly extracts traditional structural, grayscale, and electromagnetic scattering features to provide high physical interpretability, the deep learning branch is primarily expected to provide complementary local contextual feature maps rather than independently decoding all structural representations.

Additionally, the experiments were conducted on a personal computer equipped with an Intel Core i7-14650HX CPU, an NVIDIA GeForce RTX 4060 GPU, and 16 GB of RAM. The experiment used Pytorch as the deep learning framework based on Python 3.12, and CUDA12.8 was used to call the GPU for training acceleration.

### 4.3. Validation of the Effectiveness of Feature Enhancement Framework

To justify the rationality of the Retinex-based feature enhancement in the preprocessing stage of the proposed method, comparative baseline experiments were designed against alternative image enhancement frameworks, including Global Histogram Equalization (GHE) and Gamma Correction. Under identical hardware environments, the encapsulated pre-trained model utilized the ResNet34 backbone network, and the MSTAR dataset was selected for validation. Table 1 presents the performance comparison results under different feature enhancement frameworks.

To verify the effectiveness of the preprocessing mode in reducing coherent speckle noise and improving target pixel contrast, the Equivalent Number of Looks (ENL) and the Peak Signal-to-Noise Ratio (PSNR) were selected for evaluation. As quantified in Table 1, the raw unenhanced SAR patches suffer from severe phase-induced graininess, yielding an ENL of only 2.145 and a low PSNR of 14.621 dB. Implementing Global Histogram Equalization (GHE) inadvertently degrades the ENL down to 1.831; this occurs because GHE indiscriminately stretches the global pixels of the targets and misinterprets high-frequency multiplicative speckle noise as valid structural boundaries, thereby corrupting the target scattering center coordinates.

Conversely, the proposed method cascades the Improved Lee Filter with the Retinex feature enhancement, generating an exceptional ENL of 4.892 and a peak PSNR value of 22.658 dB. The Improved Lee Filter dynamically adapts its coefficients via edge detection matrices to smooth homogeneous clutter zones, while the Retinex feature enhancement effectively isolates the low-frequency illumination variations from the high-frequency reflectance maps. This demonstrates that such an architectural design can prevent the spatial representation degradation throughout the entire network, and removing or substituting this stage would directly degrade subsequent downstream classification metrics.

The experimental outcomes demonstrate that on the MSTAR dataset, the configurations implementing GHE and Gamma Correction yield F1-scores of 78.363% and 82.027%, respectively. In contrast, the integrated Improved Lee Filter and Retinex feature enhancement framework proposed in this paper achieves a superior F1-score of 84.876%. Although GHE improves macroscopic contrast by stretching pixel frequencies, it inevitably amplifies coherent background clutter and multiplicative speckle noise alongside the target scattering coordinates. Conversely, the Retinex theory successfully isolates the high-frequency reflectance component containing true structural details from the low-frequency illumination variants, this isolates true structural boundaries and localized geometry, delivering cleaner, more discriminative semantic maps to subsequent parallel extraction branches.

### 4.4. Influence of Different Pre-Trained Models on Experimental Results

As the core functional structure of the proposed method, the hierarchical feature extraction layer adopts a dual-branch hierarchical architecture and encapsulates three pre-trained models (ResNet18/34, EfficientNet-B0) in the feature extractor of the deep learning feature branch. Different models can be flexibly selected through algorithms to adapt to SAR image data.

**Figure 7:**
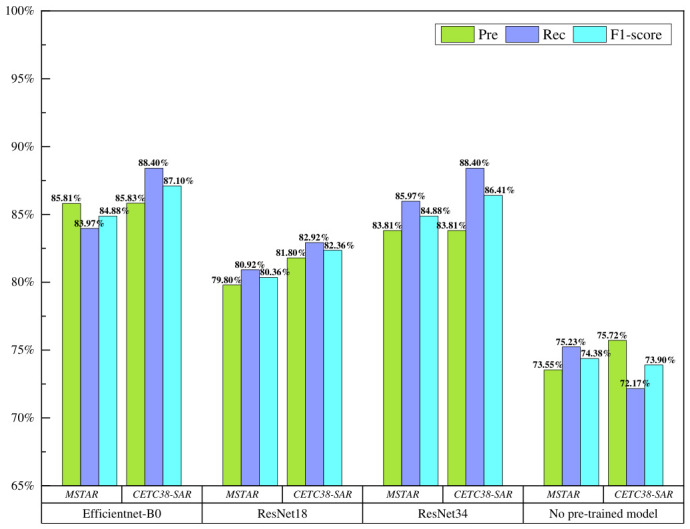
Performance comparison of the proposed method under different pre-trained models.

To verify the effectiveness of different pre-trained models on target feature extraction in the two datasets and evaluate their importance in the feature extraction process, performance experiments were conducted on the MSTAR and CETC38-SAR datasets under the same experimental settings by only changing the selection of pre-trained models. The experimental results are shown in Figure 7 , and the main indicators of the two datasets under different pre-trained models are shown in Table 2.

It can be seen from the data in Figure 7 and Table 2 that the degree of performance improvement brought by introducing transfer learning parameters varies depending on the baseline configuration and the specific data environment. Compared with the unconstrained baseline without any pretraining configuration (No pre-trained mode), the proposed method achieves a significant improvement of 5% to 15% in overall metrics under three backbone networks with packaged pretrained models. This improvement indicates that the spatial and semantic initialization obtained through pretraining helps the framework extract more reliable high-dimensional features under the influence of speckle noise.

Meanwhile, an in-depth lateral evaluation of the pre-trained backbone networks shows their performance as presented in Table 2. Both EfficientNet-B0 and ResNet34 achieved an F1-score of approximately 84.90% on the MSTAR dataset. This convergence in performance suggests that for relatively typical SAR target slices with limited shape variation, the marginal benefits of advanced structural scaling (such as the MBConv modules in EfficientNet) tend to saturate. However, when deployed on the multi-target, large-scale CETC38-SAR dataset, EfficientNet-B0 attains a higher F1-score of 87.098% compared with ResNet34 (86.047%), and ResNet34 exhibits poorer stability in precision and recall metrics. This confirms that its compound scaling strategy (such as balancing width, depth, and resolution) provides stronger robustness and higher feature differentiation thresholds only when handling scenarios filled with complex background interference.

### 4.5. Verification of Ablation Experiments

All layers of the method are connected end-to-end through data transmission to achieve target feature extraction and optimization for SAR images, providing effective information for efficiency evaluation. Under identical experimental settings, we remove the hierarchical feature extraction layer and the dynamic weighted fusion layer on the MSTAR and CETC38-SAR datasets, respectively, to investigate the effectiveness of different hierarchical structures for target feature extraction. All ablation variants maintain identical training hyperparameters and hardware environments. The detailed implementation and degradation definitions of the ablation experiments are as follows:

1. No-hierarchical feature extraction: In this variant, the parallel dual-branch architecture of traditional handcrafted features and deep learning features is eliminated. To ensure input continuity for subsequent networks, this layer degenerates into a single-branch structure that retains only the deep learning feature extraction branch. The subsequent adaptive weighted fusion layer no longer receives multi-source heterogeneous features; instead, the deep convolutional features extracted from the single branch are directly passed to the final classifier via identity mapping.
2. No-dynamic weighted fusion: In this variant, the dynamic weighted fusion layer based on dual-channel attention mechanism is removed, i.e., Equations (5)–(7) are invalidated. To evaluate the advantages of adaptive weighting, multi-dimensional features including both traditional and deep features are aligned via padding/truncation in spatial and channel dimensions, then fused using channel-wise concatenation (hard fusion) instead of dynamic weighted summation. The concatenated features are then projected to the target classification dimension via a fixed linear projection layer.
3. Proposed method: The complete framework with hierarchical feature extraction and dynamic weighted fusion is adopted.

In order to comprehensively validate the necessity of the dual-branch parallel collaboration topology, we introduced another single-branch configuration in the experimental evaluation, namely the “Traditional Baseline” variant. In this setup, only the high-dimensional deep learning feature extraction path is disabled, and the final classification layer relies entirely on the 18-dimensional physical prior features.

**Figure 8:**
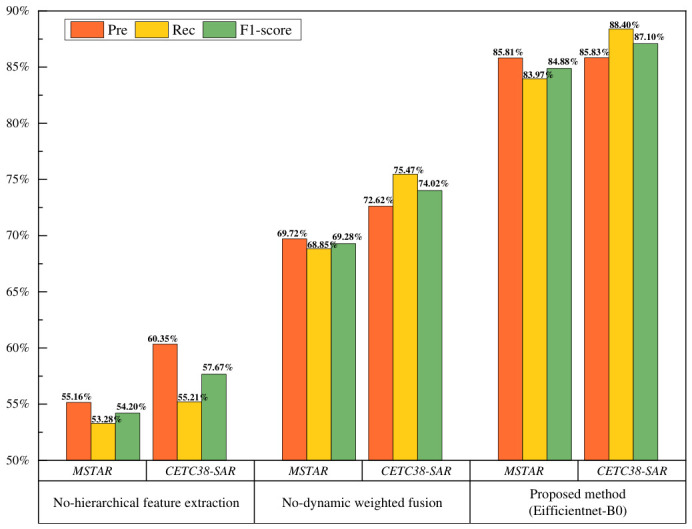
Ablation experiment based on EfficientNet-B0 pre-trained model.

**Figure 9:**
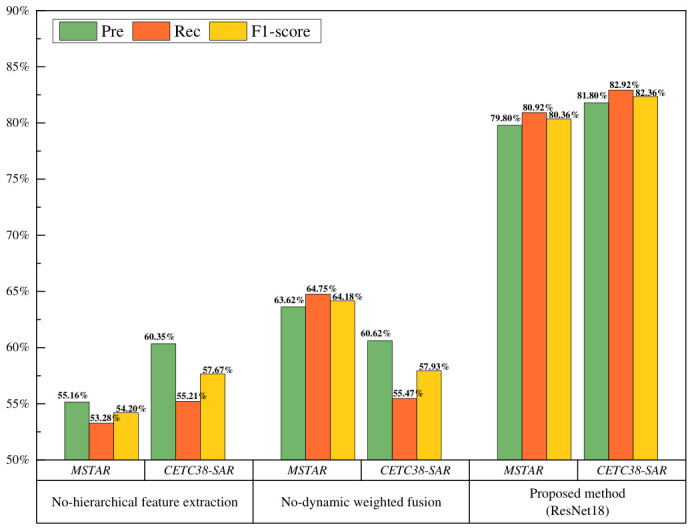
Ablation experiment based on ResNet18 pre-trained model.

**Figure 10:**
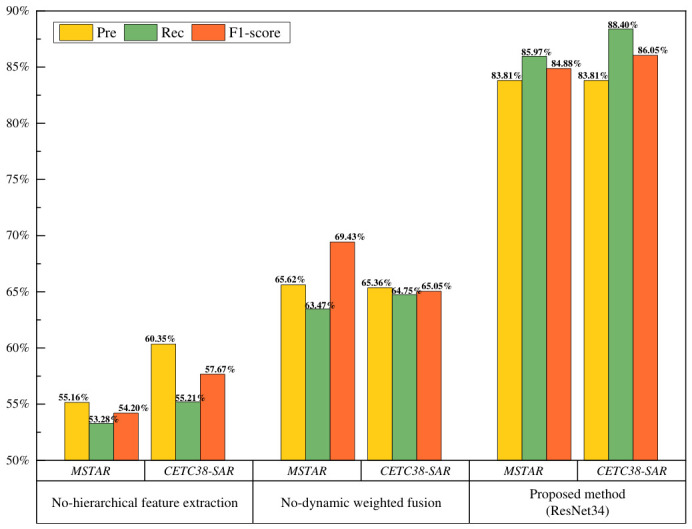
Ablation experiment based on ResNet34 pre-trained model.

**Figure 11:**
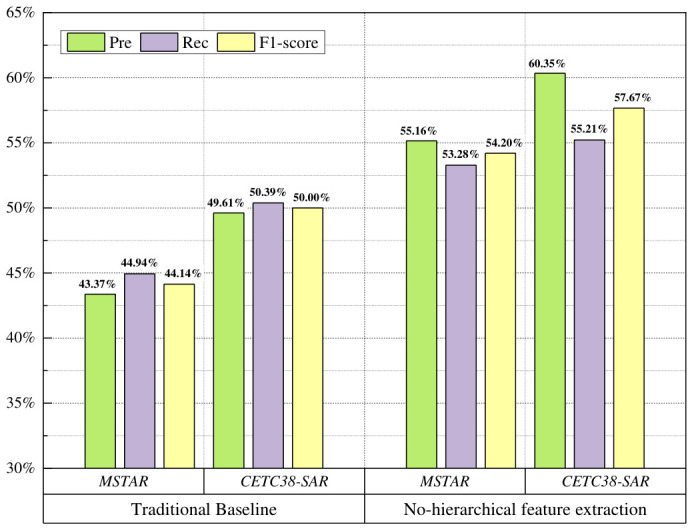
Comparison results between traditional baseline and no-hierarchical feature extraction.

The experimental results are shown in Figure 8, Figure 9, Figure 10 and Figure 11 presents a comparison between Traditional Baseline and No-hierarchical feature extraction. The main performance metrics for detecting the two types of datasets under different ablation experiment settings are shown in Table 3. In addition, to further illustrate the tensor dimension flow under different ablation configurations, taking the ResNet18 pretrained model as an example, Table 4 presents the data flow dimensions under different configuration conditions, which are used to show the design logic of the ablation experiments.

It can be seen from the data in Figure 8, Figure 9, Figure 10 and Figure 11 and Table 3 and Table 4 that all layers of the proposed method have a synergistic gain effect, and removing any layer will cause performance degradation.

Based on the ablation quantization index analysis in Table 3, when evaluating the ResNet18 baseline, after removing the feature layered extraction layer, the F1-score on the MSTAR dataset decreased significantly, from 80.356% to 54.203%, while Pre and Rec dropped to 55.156% and 53.283%, respectively. This performance degradation demonstrates that without the collaborative synergy between highly interpretable physical priors and powerful deep convolutional representations, a single-path feature extraction stream easily suffers from local overfitting when exposed to the heavy speckle noise and geometric distortions inherent in SAR images.

Similarly, when the dynamic weighted fusion layer is removed and hard concatenation fusion is adopted, the F1-score on MSTAR drops to about 64.179%, with Pre and Rec also showing significant decreases, confirming serious information redundancy and potential conflicts among multidimensional features. If hard splicing is only implemented, without attention mechanisms to provide specific enhancements in spatial and channel dimensions, and without dynamic weight $\omega_{i}^{\prime }$ adjustment contributions, useless background clutter and redundant dimensions will dilute the effective features of core scattering centers, greatly weakening the model’s generalization ability and target recognition performance in complex environments.

Meanwhile, the Traditional Baseline achieves F1-scores of only 44.140% and 49.997% on the MSTAR and CETC38-SAR datasets, respectively, which are lower than the 54.203% of the No-Hierarchical extraction (only deep learning feature extraction branch) setting on MSTAR. This demonstrates that traditional features alone are overly rigid in representing image information and fail to adapt to complex scenario variations, while the single deep learning branch lacks physical constraints and is vulnerable to noise interference. The dual-branch parallel collaboration paradigm proposed in this paper effectively balances physical interpretability and strong representation capability to a certain extent, achieving improved recognition performance in complex SAR scenarios.

In addition, by comparing the experimental results under different pre-trained models, it can be seen that regardless of the deep learning backbone network used, the performance degradation trends caused by ablation experiments on different types of datasets are highly consistent, further verifying the scientificity of the overall architecture of the proposed method, the synergy of structural layers, and the generalization ability of model design.

### 4.6. Benchmarking Against Established SAR ATR and Transformer Frameworks

To further verify the classification competitiveness and structural effectiveness of the proposed method, a horizontal comparative evaluation was conducted based on the CETC38-SAR dataset. The selected baseline models encompass A-ConvNets [38], CV-CNN [39], MSAR [40], and the latest Transformer-based object detection and recognition algorithms, while the proposed method utilizes the pre-trained EfficientNet-B0 model for comparison. Table 5 presents the performance comparison results under different algorithmic architectures.

As illustrated in Table 5, compared with existing methods, the proposed framework demonstrates stronger interpretability and target recognition capabilities. Early structural architectures (such as A-ConvNets) achieve relatively good computational efficiency; however, due to their limitations in capturing multimodal local scattering contexts, they yield a lower recognition precision of only 78.404%. Complex-valued convolutional neural networks (CV-CNN) and multi-aspect correlation networks (MSAR) deliver robust recognition performance, but they rely on rigid preprocessing pipelines or phase parameters that are frequently unavailable in unconstrained scenarios. High-capacity Transformer-based vision models capture competitive recall metrics through dense global dependency modeling, yet their massive computational overhead escalates the operational burden, making them highly susceptible to overfitting under limited data conditions. In contrast, the method proposed in this paper effectively balances these trade-offs, achieving an accuracy and F1-score of 85.831% and 87.098%, respectively, while using a lightweight architecture (approximately 6.54 M Parames). These outcomes confirm that anchoring a lightweight backbone network with explicit, interpretable domain priors can effectively mitigate the spatial representation degradation triggered by severe coherent speckle noise.

The above performance superiority mainly originates from the physically decoupled dual-branch layout and variance-adaptive fusion mechanism proposed in this work, which effectively solves the fixed-weight defect of previous dual-branch attention fusion.

### 4.7. Class-Specific Distribution and Visualization Results

**Figure 12:**
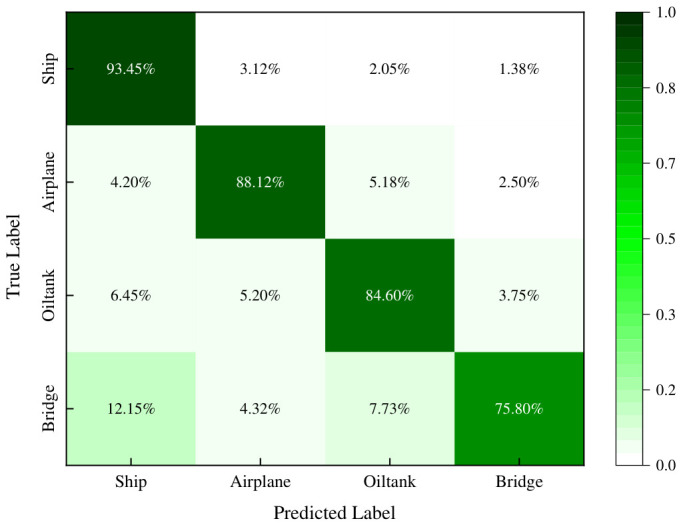
Normalized confusion matrix of the proposed method on the CETC38-SAR dataset.

Due to the class imbalance characteristic of the CETC38-SAR dataset utilized in this paper, where the number of ship targets exponentially outnumbers minority targets such as oil tanks and bridges, the macro-averaged indicators fail to adequately evaluate the recognition and classification performance of the proposed method on minority targets. To verify the recognition performance of the proposed method under varying sample sizes, Table 6 details the Precision, Recall, and F1-score of each individual category under the pre-trained EfficientNet-B0 model configuration. Concurrently, Figure 12 presents the corresponding row-normalized confusion matrix to visualize cross-class misclassification patterns.

The data streams compiled in Table 6 demonstrate that despite the severe class imbalance in the dataset, the proposed method successfully maintains high recognition sensitivity for minority targets. Specifically, the bridge target secures an acceptable F1-score of 77.09%, even though it accounts for less than 3% of the absolute training envelope. In traditional feature extraction and recognition architectures, data imbalance usually causes the optimization gradient to drift severely toward the majority class, resulting in an elevated false-negative rate for underrepresented classes. In contrast, the traditional feature branch in our method independently provides explicit physically interpretable features, and the adaptive weighted fusion layer further leverages its dual-channel attention mechanism to enhance the localized spatial features of the targets. This architectural design effectively prevents minority targets from being diluted or overwhelmed by intense background clutter and complex noise interference.

**Figure 13:**
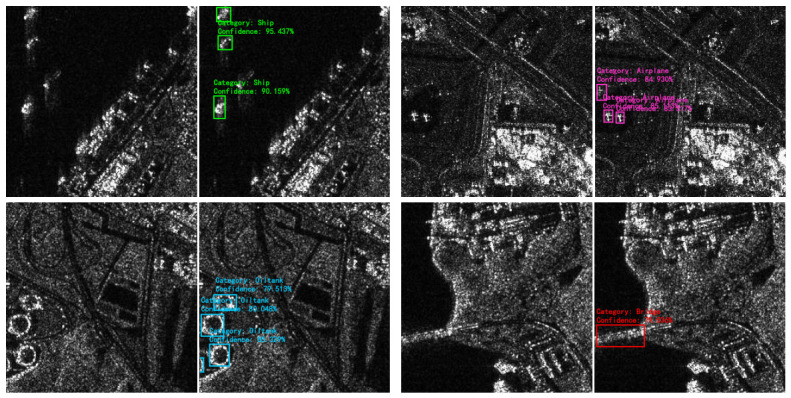
Representative target recognition and classification visualization results on the CETC38-SAR dataset.

To further enhance the intuitiveness and persuasiveness of the experimental performance, Figure 13 verbatim illustrates the representative visualization results of target recognition on the CETC38-SAR dataset.

As observed from the visual samples, high-precision localization and classification are achieved for all target categories under test. For majority targets such as ships (with a peak confidence of 95.437%) and airplanes (with a confidence of 84.930%), the network delivers highly reliable prediction results. Crucially, even when confronting minority targets substantially degraded by land clutter—such as oiltanks (with confidence scores of 79.513%, 80.048%, and 85.329%, respectively) and bridges—the model maintains stable recognition sensitivity and accurately delineates target regions. These visualization results qualitatively demonstrate that the proposed multi-dimensional collaborative architecture effectively isolates target scattering centers from complex backgrounds, thereby exhibiting superior target recognition performance.

### 4.8. Generalization and Robustness Analysis

To fully substantiate the generalization capability and algorithmic robustness of the proposed method, experimental validations were conducted under varying depression angles, progressive noise levels, and cross-dataset configurations. First, class recognition performance under variable radar depression angles was evaluated utilizing the MSTAR dataset, comparing target slices captured at a $17^{{}^{\circ}}$ baseline against alternative configurations testing variations at $15^{{}^{\circ}}$ and $30^{{}^{\circ}}$. Second, to assess anti-noise robustness, systematic noise injection trials were executed by contaminating the raw SAR validation inputs with complex additive Gaussian noise across a variance envelope of $\sigma^{2}\in \left[0.01,0.10\right]$. Finally, a cross-dataset stress test was established, wherein the proposed framework was trained entirely on the MSTAR dataset and subsequently deployed directly to interpret target features within the CETC38-SAR environment. The quantitative target recognition metrics across these extended scenario profiles are summarized in Table 7.

The empirical data profiles compiled in Table 7 demonstrate that the domain-driven physical prior knowledge embedded in the proposed method effectively alleviates the degradation of target recognition performance under Extended Operating Conditions (EOCs). When the radar depression angle shifts significantly to $30^{{}^{\circ}}$, conventional unconstrained deep architectures typically experience severe feature misalignment due to their over-reliance on the specific viewing distributions of the training set. In contrast, during the hierarchical feature extraction stage of the proposed method, the traditional feature extraction branch explicitly uncovers and extracts the geometric structures, grayscale statistics, and transform-domain identifiers of the targets to form a highly interpretable low-dimensional feature set. Because the derivation of these low-dimensional features relies heavily on explicit physics-driven mathematical formulations rather than sample abundance, the proposed method preserves a relatively stable performance threshold, despite experiencing a minor degree of metric drop.

Under intense noise contamination environments, where the raw target imagery suffers from severe phase-induced graininess, the proposed cascaded arrangement—which couples the Improved Lee Filter with the Retinex feature enhancement layer—successfully isolates the true high-frequency target reflectance from the low-frequency noise components. This mechanism systematically prevents complex background clutter from diluting the effective scattering center vectors, thereby maintaining the final recognition performance within a highly acceptable ceiling.

Furthermore, during the direct cross-dataset domain migration without any parameter retraining, the model achieves a robust F1-score of 74.460%. This strongly highlights that the adaptive dual-channel attention weighted mechanism actively prioritizes multi-dimensional feature synergies over simplistic statistical correlations, thereby establishing a highly generalizable algorithmic framework for the subsequent optimization of multidimensional feature-driven target detection and recognition methods.

### 4.9. Efficiency Performance Analysis

To verify the applicability of the proposed method in real-time situation awareness and lightweight deployment, a comprehensive evaluation of computational complexity was conducted. Based on the efficiency verification indicators configured within the SAR image target detection and recognition paradigm, Table 8 lists the total Parameter Size (Params), Floating Point Operations (FLOPs), and Inference Time across different framework backbones under identical hardware environments.

The parameters in Table 8 indicate that the multi-dimensional collaborative processing architecture proposed in this paper has good deployment adaptability and resource characteristics. When configuring the hierarchical layers with the EfficientNet-B0 backbone, the entire system requires a parameter scale of just 6.54 M (5.33 M + 1.21 M). Crucially, the model features an ultra-low computational density of 0.39 G FLOPs and Inference Time of 0.008 s/img.

Conversely, high-capacity vision models such as ViT-Base/16, which are constrained by dense global self-attention matrices mapping entire pixel sequences, burden the processing stream with a massive parameter footprint of 86.41 M and Inference Time of 0.084 s/img, imposing a heavy overall system resource overhead. In sharp contrast, the Parameter Size and Inference Time are strictly limited to 6.54 M and 0.008 s/img, respectively. This exceptional efficiency characteristics stem directly from our strategically decoupled paradigm. By transforming the task into localized slice-level patch processing, the algorithmic network concentrates its full computational representation power on the extraction of core target scattering center coordinates, entirely bypassing the need for intensive feature decoding of vast background clutter. These outcomes firmly confirm that combining explicit, low-dimensional physical domain knowledge with a lightweight learning backbone achieves an optimal trade-off between categorization accuracy and edge-device deployment feasibility.

## 5. Conclusions

This paper constructs a dual-branch multi-dimensional adaptive feature fusion framework combining physically interpretable handcrafted features and deep convolutional features to tackle speckle noise interference, geometric distortion and insufficient feature fusion efficiency existing in conventional SAR target recognition algorithms. Comprehensive quantitative experiments on MSTAR and the public CETC38-SAR datasets validate the effectiveness and robustness of the proposed approach.

First, the cascaded Improved Lee filtering and Retinex enhancement preprocessing significantly suppress SAR multiplicative speckle noise. Compared with GHE and Gamma correction preprocessing schemes, the proposed preprocessing improves the dataset’s average F1-score by 6.513% and 2.849% respectively on MSTAR, raising the image PSNR to 22.658 dB and effectively purifying target scattering feature information for subsequent feature extraction.

Second, the dual-branch hierarchical extraction design realizes complementary advantages of low-dimensional physical features and high-dimensional deep features. Ablation results verify that removing either traditional feature branch or deep feature branch leads to an obvious F1-score decline exceeding 20% on both datasets, which proves physical prior constraints effectively reduce the risk of network overfitting under small-sample SAR conditions.

Third, the variance-driven dual-channel adaptive weighted fusion outperforms fixed concatenation hard fusion. With EfficientNet-B0 as the backbone, the proposed method achieves 85.831% precision and 87.098% F1-score on CETC38-SAR, surpassing A-ConvNets, CV-CNN and Swin-Transformer mainstream baselines while controlling total model parameters at only 6.54 M and single-image inference time as low as 0.008 s. The lightweight property guarantees deployment feasibility on embedded airborne SAR terminal devices.

Fourth, the proposed framework exhibits strong generalization under extended working conditions: under $30^{{}^{\circ}}$ depression angle deviation, severe Gaussian noise ($\sigma^{2}=0.10$) and cross-dataset transfer from MSTAR to CETC38-SAR, the model still maintains Precision higher than 71%, and achieves acceptable recognition accuracy for scarce samples such as bridges (F1-score = 77.09%) amid serious category imbalance, showing prominent practical value for battlefield irregular target identification.

In practical engineering, the decoupled preprocessing-feature-fusion architecture can be directly embedded into airborne/shipborne real-time SAR reconnaissance systems to improve clutter suppression and target classification reliability. Nevertheless, the algorithm still has deficiencies for ultra-low SNR SAR scenes and ultra-small dim targets. Future research directions include further lightweight backbone optimization, multi-polarization feature excavation and few-shot learning improvement to expand the algorithm’s practical application scope under complicated all-weather SAR reconnaissance scenarios.

## Figures and Tables

**Table 1:** Performance metrics under different feature enhancement frameworks.

Removed Layer	ENL	PSNR (dB)	Pre (%)	Rec (%)	F1-Score (%)
No-feature enhancement	2.145	14.621	65.038	69.752	67.313
Global Histogram Equalization (GHE)	1.831	12.105	77.224	79.536	78.363
Gamma Correction (γ=1.5)	2.573	16.218	80.715	83.382	82.027
Enhancement Based on Retinex Features	4.892	22.658	83.813	85.967	84.876

**Table 2:** Main performance indicators of target detection on two datasets under different pre-trained models.

Dataset	Pre-Trained Model	Pre (%)	Rec (%)	F1-Score (%)
MSTAR	ResNet18	79.797±1.23	80.923±1.56	80.356±1.32
	ResNet34	83.813±1.05	85.967±0.98	84.876±1.01
	EfficientNet-B0	85.813±0.82	83.967±0.85	84.880±0.88
	No pre-trained model	73.547±2.15	75.233±1.98	74.380±2.03
CETC38-SAR	ResNet18	81.797±1.15	82.923±1.38	82.356±1.21
	ResNet34	83.813±0.96	88.403±1.02	86.407±0.99
	EfficientNet-B0	85.831±0.68	88.403±0.63	87.098±0.54
	No pre-trained model	75.722±1.78	72.166±2.05	73.901±1.96

**Table 3:** Main performance metrics of two types of datasets detection under different ablation experiment settings.

Removed Layer	Dataset	Pre (%)	Rec (%)	F1-Score (%)
Traditional Baseline	MSTAR	43.372±3.47	44.935±3.28	44.140±3.15
	CTEC38-SAR	49.613±2.78	50.386±2.69	49.997±2.93
Proposed method (ResNet18)	MSTAR	79.797±1.23	80.923±1.56	80.356±1.32
	CTEC38-SAR	81.797±1.15	82.923±1.38	82.356±1.21
No-hierarchical feature extraction	MSTAR	55.156±3.41	53.283±2.83	54.203±3.05
	CTEC38-SAR	60.350±2.36	55.215±2.31	57.668±2.27
No-dynamic weighted fusion	MSTAR	63.621±1.97	64.746±2.11	64.179±2.52
	CTEC38-SAR	60.621±2.06	55.475±1.58	57.934±2.14
Proposed method (ResNet34)	MSTAR	83.813±1.05	85.967±0.98	84.876±1.01
	CTEC38-SAR	83.813±0.96	88.403±1.02	86.047±0.99
No-hierarchical feature extraction	MSTAR	55.156±3.41	53.283±2.83	54.203±3.05
	CTEC38-SAR	60.350±2.36	55.215±2.31	57.668±2.27
No-dynamic weighted fusion	MSTAR	76.621±1.55	63.475±1.83	69.431±2.01
	CTEC38-SAR	65.362±1.34	64.746±1.77	65.053±1.81
Proposed method (EfficientNet-B0)	MSTAR	85.813±0.82	83.967±0.85	84.880±0.88
	CTEC38-SAR	85.831±0.68	88.403±0.63	87.098±0.54
No-hierarchical feature extraction	MSTAR	55.156±3.41	53.283±2.83	54.203±3.05
	CTEC38-SAR	60.350±2.36	55.215±2.31	57.668±2.27
No-dynamic weighted fusion	MSTAR	69.721±1.17	68.847±1.42	69.281±1.53
	CTEC38-SAR	72.621±1.09	75.475±1.32	74.020±1.38

**Table 4:** Data stream dimensions under different configuration conditions.

Configuration	Hierarchical Output Dim	Fusion Operation	Fused Feature Dim	Classifier Input Dim
Traditional Baseline	$F_{trad}\in R^{18}$	N/A	$R^{18}$	$R^{18}$
No-hierarchical feature extraction	$F_{deep}\in R^{512}$	Identity Pass	$R^{512}$	$R^{512}$
No-dynamic weighted fusion	$F_{trad}\to^{512}$ $F_{deep}\to R^{512}$	Concat + Linear Layer	$R^{1024}\to R^{512}$	$R^{512}$
Proposed method	$F_{trad}\to^{512}$ $F_{deep}\to R^{512}$	Dual Attention + Weighted Sum	$R^{1024}\to R^{512}$	$R^{512}$

**Table 5:** Performance indicators of different algorithm architectures.

Methodology Paradigm	Core Backbone Network	Pre (%)	Rec (%)	F1-Score (%)	Params (M)
A-ConvNets	Custom All-CNN	78.404	79.156	78.778	0.35
CV-CNN	Complex-Valued Block	81.257	80.598	80.926	2.14
MSAR	Multi-Aspect Fusion	82.330	81.904	82.116	8.45
Transformer-based Framework	Swin Transformer [22]	84.105	85.268	84.682	22.40
Proposed method	EfficientNet-B0	85.831	88.403	87.098	6.54

**Table 6:** Recognition performance of various targets on the CETC38-SAR dataset.

Target Category	Total Samples	Sample Ratio (%)	Pre (%)	Rec (%)	F1-Score (%)
Ship	13,038	64.52	91.24	93.45	92.33
Airplane	5699	28.20	86.50	88.12	87.30
Oiltank	919	4.55	82.15	84.60	83.36
Bridge	551	2.73	78.43	75.80	77.09

**Table 7:** Target recognition performance under Extended Operating Conditions (EOCs).

Evaluation Domain Type	Test Profile Specifics	Pre (%)	Rec (%)	F1-Score (%)
Depression Angle Shift	SOC ($17^{{}^{\circ}}$ baseline)	85.813	83.967	84.880
	EOC-1 ($15^{{}^{\circ}}$ variant)	84.105	82.314	83.200
	EOC-2 ($30^{{}^{\circ}}$ variant)	79.421	76.508	77.937
Noise Contamination	Mild Noise ($\sigma^{2}=0.01$)	83.142	81.339	82.231
	Moderate Noise ($\sigma^{2}=0.05$)	79.204	77.116	78.146
	Intense Clutter ($\sigma^{2}=0.10$)	71.458	68.312	69.805
Cross-Dataset Migration	MSTAR→CETC38-SAR Transfer	75.127	73.804	74.460

**Table 8:** Computational complexity metrics of different framework backbone networks.

Framework Backbone Network	Params (M)	FLOPs (G)	Inference Time (s/img)
ResNet18	11.69 + 1.21	1.82	0.012
ResNet34	21.28 + 1.21	3.67	0.019
EfficientNet-B0	5.33 + 1.21	0.39	0.008
Baseline ViT (ViT-Base/16)	86.41	17.64	0.084

## Data Availability

The data presented in this study are available on request from the corresponding author due to privacy.
